# The recovery processes among paramedics who encountered violence during work—a narrative interview study

**DOI:** 10.1186/s12995-024-00417-6

**Published:** 2024-05-16

**Authors:** Veera Kamaja, Hilla Nordquist

**Affiliations:** 1 Jokilaakson Terveys Oy, Sairaalantie 11, Jämsä, 42100 Finland; 2 Department of Healthcare and Emergency Care, South-Eastern Finland University of Applied Sciences, Pääskysentie 1, Kotka, 48220 Finland

**Keywords:** Emergency medical service, Paramedic, Workplace violence, Recovery process

## Abstract

**Background:**

Almost all paramedics encounter workplace violence (WPV) during their careers. The most common form of WPV is verbal, and the perpetrator is usually the patient. It is known that paramedics suffer from post-traumatic stress disorder and other mental health problems, and WPV is one of the reasons behind that. Nevertheless, little is known about the recovery processes paramedics have had after encountering WPV. The research question was: What kind of recovery processes have paramedics had after encountering WPV?

**Methods:**

A qualitative, narrative interview study was done. Data was collected in individual interviews with Finnish paramedics (*n* = 18). Paramedics were from different parts of Finland, and their ages varied from 24 to 49 years. They had been working in EMS for an average of 10.5 years (range 1.5 to 25 years). Interviews were conducted with a narrative approach, which enabled paramedics to narrate their experiences and speak on their own terms about the subject to the extent of their choosing. The data was analyzed using thematic analysis.

**Results:**

Ten recovery process themes were identified: Strong psychological and physical reactions in a short time frame, Questioning one’s profession and actions, Various support structures aided in recovery, Dysfunctional processes hindered recovery, Personal resources provided support, The support of the workcommunity as a lifeline, Left to cope alone, Permanent changes to work routines, Resulting in professional growth and Eternal crack in the shell.

**Conclusions:**

Many internal and external factors affect paramedics’ recovery processes. While some receive adequate help, others struggle to get appropriate support, especially from their organization and supervisors. The findings of this study suggest that clear protocols should be established to help paramedics recover after encountering WPV and that an individual aspect should be kept in mind, as not everybody reacts in the same way.

**Supplementary Information:**

The online version contains supplementary material available at 10.1186/s12995-024-00417-6.

## Introduction

Paramedics are healthcare professionals who work in Emergency Medical Services (EMS), doing demanding work while attending various types of missions outside of the hospital. Missions vary from ambiguous symptom patterns and cardiac arrests to violence-related calls and, nowadays, more often, to diverse sets of mental health issues [1–4]. In Finland, paramedics operate mainly in paramedic-staffed units. Additional EMS units, including a physician-staffed ground unit or helicopter EMS crew, join only the most critical missions. The work field in EMS is challenging, as paramedics work mainly in pairs in people´s homes, in crowds, and inside cramped ambulances, making the work environment very unpredictable.

The diverse setting of paramedics´ work field increases the risk of facing workplace violence (WPV), and thus, most of the paramedics have encountered WPV during their careers [5–8]. The most common form of WPV is verbal [6–9], and the perpetrator is usually the patient [5, 8, 10]. Incidents are underreported [8–12], partly because paramedics consider them part of their work, policies are unclear, and paramedics may find filing a report as a sign that they failed to handle a mission [11]. Immediately after facing WPV, paramedics have reported feelings of despair, humiliation, anger, frustration, insecurity, fear, irritability, defensiveness, anxiety and detachment, and also physical reactions such as disrupted sleep [7, 12–14]. Experiencing WPV increases the prevalence of depressive disorders [15] and symptoms of stress, anxiety, depression, and burnout significantly [5, 8, 9, 16, 17]. Experienced WPV is associated with poor mental health [18] and with an increased risk of suicide attempts or death by suicide [19]. Post-traumatic stress disorder is more common amongst paramedics compared to the general population, and WPV is one of the reasons for that [6, 8, 16, 20–22]. It may also reduce paramedics’ job satisfaction levels [7, 23, 24]. Furthermore, there is some evidence that experiencing WPV may negatively affect the quality of patient care [8, 23, 24].

EMS organizations should have policies for mitigating the risk of WPV and addressing WPV incidents [25, 26]. A systematic review by Drew et al. (2020) demonstrated the importance of these policies being tailored to the specific cultural, environmental, and social factors of each organization, and that the continuous evaluation of these policies is crucial. These policies could include, for example, a system-wide commitment and response to violence, clear alignment of WPV, and different types of education and training for the paramedics (e.g., use of physical restraints, self-defense skills and body-language assessment) [26]. An Iranian study recommended training in stress management, familiarizing the community with the responsibilities of EMS, reducing response time, creating efficient communication systems, using appropriate facilities and enough personnel, training violence control, improving employees’ job satisfaction, and coordinating cooperation with the police to reduce WPV [27]. Additionally, training to observe early warning signs before anything happens is important and can reduce the occurrence of violent incidents [26, 28, 29]. Gillespie et al. (2010) proposed several protective strategies for protecting against the negative consequences of WPV. These strategies include personal protection (taking action against the perpetrator), fostering healthy self-support mechanisms, receiving support from others, and changing current practices [30]. It is evident that paramedics would benefit from receiving training concerning needed skills to cope with and recover from critical incidents or WPV [25, 31].

How people react to and recover from traumatic incidents, including WPV, depends on many factors, for example, on the cumulative effects of intellectual abilities, interpersonal capabilities, creativity, productivity, and the ability to manage, change and respond to diversity [25]. In addition, it is not uncommon for a person to have encountered multiple cases of traumatic incidents or WPV and only require assistance after the latest one after nearly exhausting their coping skills [25]. Two concepts arise in previous studies: resiliency and resistance. Resilient people are able to maintain their relatively stable levels of functionality even after encountering an extremely stressful incident, such as WPV [25, 32, 33], and resistance can be described as a form of psychological immunity to distress and dysfunction [33]. Both of these can be fostered by certain strategies, such as fostering group cohesion and social support and building self-efficacy [33], thus being something organizations should look into. Having a high level of resiliency can also be a protective factor against WPV, which could be attributed to the fact that resilient people tend to respond to stressful situations in positive and constructive ways, thus promoting healthy behavior in others [32].

Although WPV may have many consequences for paramedics, little is known about how paramedics experience the incident itself, its aftermath, and their recovery processes. As noticed in previous studies [6, 14, 17, 22, 23, 34–39], WPV has the potential to be a significant stressor not only in their working life but private life as well, affecting paramedic´s lives thoroughly and potentially for an extended period. Therefore, in this study, we aimed to explore how paramedics describe their recovery processes after WPV. The research question was: What kind of recovery processes have paramedics had after encountering WPV? The results are useful in informing the design of appropriate measures to prevent and handle violent episodes and their aftermath.

## Materials and methods

This study was performed as a qualitative interview study with paramedics, using an approach based on narrative health psychology [40]. This narrative approach allowed the participants to share their personal stories about their experiences with health and illness, specifically their recovery processes after experiencing WPV.

### Study sample and recruiting procedure

Participants were recruited via closed Facebook groups that were for EMS professionals in Finland. A research announcement was published in two groups in May 2023, and paramedics who met the inclusion criteria were asked to contact the first author by email if they were interested. The inclusion criteria were: (1) an adult who is or has been working as a paramedic for at least a year, (2) had experienced workplace violence or threat of it while working in EMS, and (3) had fully recovered from the incident. In this study, “fully recovered” meant that participants felt they had fully recovered from the incident and were able to discuss the subject without strong emotions. During the recruitment process, the interviewer ensured that participants had processed the incident and recovered from it. In addition to the Facebook announcement, a snowball method was used to recruit participants. At the end of the interviews, participants were asked whether they knew others who met the inclusion criteria. If they did, the first author contacted those persons by email and provided them with information about the study. In addition, the first author provided information by email about the study for paramedics who were known for encountering workplace violence. All interested/potential participants, regardless of the recruitment method, were first provided with a participant information statement and privacy statement of the study, and they were given an opportunity to ask questions and decline before signing a written informed consent to participate.

The recruitment of participants lasted eight weeks between May 2023 and July 2023. By the end of the recruitment period, 18 participants were found and interviewed, as the interviews were conducted soon after participants were enrolled. All participants had encountered workplace violence, though the violent incident itself or type was not asked about, as it was not seen as relevant to this study.

### Data collection

Data was collected by interviewing participants individually via Microsoft Teams, using a narrative method. The first author was the interviewer. She is an experienced advanced-level paramedic, and at the time of the research process, she was pursuing further studies under the guidance of the last author. A pilot interview was completed, and it was later included in the study as the participant gave their agreement. The interview was found to be successful, as relevant information was revealed by using the narrative interview method and strong emotions did not occur. Only one participant and the first author were present during the interviews. Each participant was interviewed once and, as per the narrative approach [40], had the opportunity to talk about the subject in their own words, answering only the question, “Would you tell me the story of your recovery process after the workplace violence you experienced?” [41] The interviewer asked clarifying questions as necessary [41], such as “Would you tell me more about your feelings during that time?” or “Could you describe that a little more?” Background information was also asked, including age, gender, level of professional qualifications (basic/advanced), the region of Finland they were working in, whether it was rural or urban, years of working experience, and time since the incident. The audio was recorded, and Microsoft Teams transcription was used during the interviews. Interviews lasted from 17 to 69 min (an average of 39 min) and were conducted from May to July 2023. The transcriptions were checked and completed manually, and the data was pseudonymized at this point by removing any identifying information mentioned during the interviews. The whole transcript consisted of 46,605 words (excluding background information). Transcripts were not sent to participants for comments.

### Data analysis

In this study, the data was analyzed with thematic analysis [41–43], emphasizing the content of the narratives and allowing for the finding of common thematic elements across the recovery narratives of the participants [43]. This analysis method was chosen because multiple adjacent recovery processes arose from each interview. Narratives were analyzed, preserving their uniqueness and exact wording [41, 43] and identifying for themes that the narratives portrayed [41]. Analysis was inductive—recovery process themes were allowed to rise from the material instead of searching for themes that would correspond with prior research evidence or theories [41, 42].

At the beginning of the analysis process, the first author familiarized herself with the data, read through it several times, and made preliminary notes on each story. Narrative sections that corresponded to the research question were selected as units of analysis [41]. Then, she identified those narratives from the data, copied them to another Word document and organized them under preliminary headings describing the narrative content. After this, similar narratives were organized as in thematic analysis and named in accordance with the content [42]. The recovery processes identified were not always temporally precise and sequential, as they could overlap and occur in different areas of life. All of the themes represent a phase of recovery, which had distinguishing features from other described recovery processes. The whole analysis was an iterative process [41, 42] and was done in close discussion with the last author, who is an experienced senior researcher. When both authors agreed on the results of the analysis, narratives from the original data were translated into English, trying to preserve their vividness and accuracy as well as possible. The results of the analysis are presented as recovery process themes, with their content explained in text and original quotes from the interviews. Due to limited space, further quotes are presented in Appendix 1.

### Ethical considerations

The research considers paramedics’ experiences and recovery after encountering workplace violence, a rather sensitive subject. The good scientific practices and ethical principles of research with human participants [44, 45] were strictly followed. As the Finnish National Board on Research Integrity TENK emphasizes, the fundamental starting point for research with human participants is the participants´ trust in researchers and science. This trust is to be preserved throughout the research process. It is obligatory to respect the human dignity and rights of the research participants. The layout of this study was such that it could have elicited various emotional reactions from participants. Mild mental strain was acknowledged to be allowable, but it should not have exceeded the limits of everyday life situations, as required by TENK. Participants were informed about this risk in advance. In order to avoid causing them unnecessary harm, attention was paid to ensuring all participants had processed the incident and fully recovered. Each participant was clearly asked whether they had fully recovered from the incident(s).

Participation was voluntary and based on informed consent. The first author offered information about the research to potential participants so that they had a realistic understanding of it in advance and could decide for themselves if they wanted to participate. The authors did not insist that anyone participate.

During interviews, participants had the right to talk about the subject in their own words, at a level they felt safe and confident. The interviewer ensured that the atmosphere and situation were peaceful and that outsiders could not hear the interviews. Participants had the right to withdraw their consent at any time during the research. No dropouts occurred during the study.

## Results

Of the 18 participating paramedics, 12 were women, and 6 were men, ages varying from 24 to 49 years. Participants were from different regions of Finland and were working both in rural and urban areas. They had been working in EMS for an average of 10.5 years (range 1.5 to 25 years). Seventeen participants were working at an advanced level, and one was working at a basic level. Seventeen of them were still working as paramedics.

Ten recovery process themes were identified: Strong psychological and physical reactions in a short timeframe, Questioning one’s profession and actions, Various support structures aided in recovery, Dysfunctional processes hindered recovery, Personal resources provided support, The support of the work community as a lifeline, Left to cope alone, Permanent changes to work routines, Resulting in professional growth and Eternal crack in the shell.

### Strong psychological and physical reactions in a short timeframe

Experiencing workplace violence caused, for most of the interviewees, intense psychological and physical symptoms, which started in a short timeframe after the incident. For some, the symptoms lasted from a few days to a few weeks, while for others, they persisted for months. Generally, the most severe symptoms began to alleviate within days. Interviewees reported how the incident did not fully register in their consciousness until they changed out of their uniforms into civilian attire or when they arrived home. Getting home was not taken for granted, as expressed by interviewee I:

*“[…] and at that point, it was a powerful and overriding feeling that it is not at all obvious that I am driving home now. […] And then I remember that I was quite teary at home that day, too, and somehow it was a bit like a shock reaction from that. And even then, everything was related to the fact that when I got home to my children, it was not obvious at all that I had made it.”*

In the following days, interviewees described experiencing symptoms such as insomnia, palpitations, and memory problems. Various states of fear emerged—the need to peek over one’s shoulder and repeatedly check that the front door was locked. Some feared the person who had committed the violence would seek revenge, while others had more generalized fear, resulting from a breach in their sense of security. (For example, interviewee M describes the fear in Appendix 1: Quote 1).

Psychological stress significantly affected the interviewees’ daily civilian life, as the incident constantly occupied their minds, making concentration difficult. Descriptions included feelings of irritability, frustration, and emotional sensitivity, as described by interviewee B:

*“For the first three days, I was incredibly prone to crying, so I cried over every little thing. If something didn’t go well, it frustrated me immensely, like when there’s chaos at home, nothing can be found, and you try to prepare some food, and the knives are in the wrong place. And then I have a kind of island in the kitchen, which is full of stuff, so there’s not enough space to cook properly. So, at one point, I thought about throwing dishes out of the window, like whatever, I can just go and get some cheap dishes from IKEA to replace them, like I don´t have the energy to care, like couldn’t things just go smoothly? So, I was very frustrated, incredibly frustrated, and tearful. I could just burst into tears at any moment. I couldn’t really concentrate on anything.”*

For most, the symptoms eased quite quickly in civilian life but worsened upon returning to work. Returning to work, either for the next scheduled shift or after sick leave, caused anxiety, even during the night before. Once at work, interviewees reported feeling tense but were still able to perform their duties well. (For example, interviewee C described how their state stabilized when they immersed themselves in everyday activities at work. Appendix 1: Quote 2).

### Questioning one’s profession and actions

The unexpected experience of workplace violence led the interviewees to question their profession and actions. Paramedics faced thoughts of whether they wanted or were capable of doing a job in which such incidents could occur. (For example, interviewee P described questioning their thoughts, which reinforced the idea of seeking a change in their situation. Appendix 1: Quote 3).

There was a lot of “what if” thinking—what could have happened, what potential outcomes the situation held. Thoughts revolved around the incident. Even if they had survived the situation without physical harm or with minor physical injuries, the interviewees had to contemplate very severe scenarios, as described by interviewee Q:

*“And, like, if I hadn’t managed to break free from that stranglehold, what could have happened then? Would I have been out cold on the floor, and would I have lost my life? I started thinking about things like that.”*

They received help and validation for their uncertain thoughts and questioning from either conversations with coworkers or reasoned themselves that they had reacted as well as possible given the situation. Paramedics also pondered how close it had been for the situation to have potentially escalated. Many summed it up to bad luck. (For example, interviewee K described their own experiences in Appendix 1: Quote 4).

The interviewees also reasoned that such situations could not have been prevented practically. Often, the individuals causing the threat and violence were in a mentally unstable state and not entirely in touch with reality, making their actions unpredictable. Approaching the situation professionally and analytically eased paramedics’ feelings when they were able to rationalize the situation. (For example, explanation by interviewee F in Appendix 1: Quote 5).

The interviewees also raised concerns about whether they would be able to handle similar situations in the future. (For example, thoughts of interviewee R in Appendix 1: Quote 6).

### Various support structures aided in recovery

Many of the interviewed paramedics benefited from various support structures in their recovery. They did not have to face their experiences and feelings entirely alone but received external assistance in processing the situation. Some of them received formal support from their employer, while others sought formal help on their own. The interviewees used services and measures such as occupational health services, supervision of work sick leave, and defusing sessions (defusing sessions are discussions led by a trained peer and usually arranged within 24 h after a WPV or critical incident).

Soon after the incident, at the latest on the following day, officially organized defusing sessions, structured peer support, or informal discussions brought relief to many. In these sessions, the threat and violence situation were discussed step-by-step chronologically, various possible emotional reactions were addressed, and information about seeking help was provided. The sessions normalized reactions and helped the interviewees make sense of what had happened. (For example, interviewee B described their thoughts in Appendix 1: Quote 7).

Interviewee I highlighted the importance of the defusing session leader in destigmatizing the situation and giving allowance for the shock:

*“But then on the way home, at that point, we had just started this threat and violence support, well, it was like an experiment that they are always called through. Then I called one of those people who conduct these sessions. I asked if they could defuse with me now, and then we talked on the phone during the journey home and defused the incident.*

*And they normalized my feelings about it very well because I initially had thoughts like, ‘I cannot have these feelings. Why do I feel like this when nothing happened? Like there was nothing really to it?’ So they kind of, maybe, destigmatized it rather than normalized it. That no, it is not normal for you to face such things. They are not normal to us. During that call, I understood quite well that, for example, when it comes to a weapon, if a person threatens you with a knife, a knife is a relatively common thing for us. We do cut vegetables and such, so it is a little bit more ordinary, but a normal person doesn’t deal with weapons. So, it immediately adds a scarier element just because it is unfamiliar. But that call was good.”*

The interviewees received assistance from occupational health services, such as sick leave and sleeping aids. The most common form of assistance mentioned was visits to occupational health psychologists. The psychologist’s expertise and understanding of the paramedics’ work environment were critical for the interviewees in terms of the usefulness of the assistance. (For example, interviewee J described benefiting from these visits in Appendix 1: Quote 8).

In addition to therapy sessions, one interviewee received eye movement desensitization and reprocessing therapy (EMDR). (For example, interviewee C found this form of treatment very effective and beneficial for their recovery, as they stated in Appendix 1: Quote 9).

Many of the interviewees were on sick leave for a short period following the incident. Interviewees felt that short-term sick leave was necessary after a traumatic incident but also pointed out that prolonged absence could create a barrier to returning to work. Interviewee (P), however, felt that they specifically benefited from an extended leave of absence, made possible by taking educational leave (A statutory right to take voluntary leave from work to accomplish studies).

*“In a way, for me, there was relief in that I went on leave quite quickly after the incident. So, I initially started my vacation, and then the educational leave began. […] I was already on vacation, and I would have had a couple of weeks of work before the start of educational leave. So I then informed that I was not coming back to work, that I still had unused vacations or days off, and that I was going on vacation now, and then I was not coming back to work before X. So, we managed that very well, and I feel that what has made this process easier for me was that I was away from work for a long time and that I, like, got to process the matter myself and then find the good things about my profession, what the real reasons are why I do this work.”*

### Dysfunctional processes hindered recovery

Many paramedics faced challenges in their recovery that were beyond their control. Sometimes, their need for help was not recognized in a timely manner, or the assisting party was not qualified to handle someone who had experienced a traumatic incident. Negative experiences eroded their trust in those offering help. Interviewee D described how roles seemed to reverse during a visit to a psychologist:

*“I went to an occupational health psychologist, but it was, well, it was quite a funny visit because this occupational health psychologist was absolutely shocked that, oh my god, in your work, this can happen, so it was like a legendary ‘paramedic meets psychologist’ type of situation, like, well, ‘Do I need to comfort you?‘—kind of. So, it was somewhat like, I am here because I can process this incident, and I know that it will ultimately benefit me. But yeah, the benefit was greatly diminished because the psychologist, well, I had to explain to them what our work is, what this situation was, and how something like this can happen. It involves a lot of, in a way, unpacking the situation as a whole, too, because I had to explain to someone else what it was all about.”*

Several interviewees reported experiencing multiple incidents of workplace violence within a short period. The first incident was often dismissed to some extent and not recognized as a burden that needed to be addressed and processed. The second incident increased mental strain to the point where the breaking point was reached. (For example, interviewee B decided that a break from work was needed, as they described in Appendix 1: Quote 10).

If the incident led to a criminal legal process, it almost always prolonged the recovery process for those involved. Court proceedings could take place six months or a year after the incident, causing traumatic experiences to resurface. During the legal process, paramedics were exposed to different kinds of stress; the situation was new to many, and the bureaucracy was unfamiliar. (For example, interviewee F was not prepared for the stress caused by the legal proceedings, as they reported in Appendix 1: Quote 11).

Questioning by the opposing attorney, even though it is part of their role, felt unfair to paramedics who had experienced the incident due to their work. In court, the incident that occurred at work could be treated as an action against a civilian, and paramedics often found themselves alone in court. Interviewee O described the court proceedings as an almost absurd experience. During the trial, the defense attorney made harsh accusations against the paramedic’s actions and tried to turn the incident into a dispute between two civilians. Due to privacy concerns, the exact quote is not displayed.

Dysfunctional processes could also refer to defusing session arrangements. One interviewee was placed in a difficult position because they had to organize a defusing session for other people, even though they had also been involved in the traumatic situation themself. (For example, in Appendix 1: Quote 12, Interviewee H described how their recovery process was delayed as a result).

### Personal resources provided support

Paramedics described various personal resources, skills, expertise, and abilities to cope with a distressing incident. Many interviewees highlighted their professional knowledge and how it helped them understand the different phases of a crisis and various physical and psychological reactions. (For example, interviewee D felt that their background was the most significant asset in recovery, as they described in Appendix 1: Quote 13).

They knew that they could move forward by talking about what had happened. Personal readiness and qualities aided their progress. Interviewees emphasized both a gentle attitude toward themselves and the importance of self-reflection. Interviewee I described their mental strengths in the recovery process as follows:

*“Maybe the kind of self-reflection and openness, and of course, having people close to you with whom you can talk about things, and also maybe courage, the courage to recognize your feelings and the courage to say out loud that if something is scary or felt bad or unpleasant or made you anxious or something, so that kind of openness, the close circle of people, those factors definitely [helped me move forward].”*

Life experience, both the wisdom that comes with age and knowledge gained through work experience, was recognized as a strength. Many had experienced several threats and violent incidents in their careers, but not all of them had led to strong reactions. However, some incidents were perceived as different and significantly more burdensome, often for reasons that interviewees could not pinpoint. Previous experiences both increased the burden and lightened it over time. (For example, interviewee O, with extensive work experience, described how diverse experiences had facilitated their recovery in Appendix 1: Quote 14).

In terms of personal support resources, interviewees described that consciously focusing on pleasant things, such as hobbies that brought joy, diverted their thoughts from the incident. (For example, interviewee Q explained how their personal interests aided his recovery in Appendix 1: Quote 15).

Not all paramedics had the personal resources to go through the legal process, but the ones who did also derived strength and solace from the process; their ability to progress through the process provided them with endurance, and seeing the perpetrators punished for their actions felt meaningful and brought a sense of satisfaction. (For example, interviewee D shared experiences regarding this in Appendix 1: Quote 16).

### The support of the work community as a lifeline

The support of the work community has been an essential factor for the interviewees in moving forward. At work, they had the permission to react and the freedom to talk about the threats and violence they had experienced. Interviewee B felt that the entire work community supported them.

*“It really touched me a lot that […] that the work community supports you, that they are genuinely concerned, and they send messages afterward. For instance, right after the incident, a coworker sent a message saying hey, doughnuts are waiting for you at the station. They made sure that, like, another coworker said, ‘On Wednesday, you are coming to get some paw therapy, and we are not discussing this anymore, period.’ So, it’s like that, like when everyone cares about you. I was not used to that at all.”* (Author comment: In this context, paw therapy means petting dogs.)

The workplace itself felt like a safe environment, even though going to work or responding to emergency calls caused anxiety. Others in the field understood the realities of such situations. (For example, interviewee C considered the support of the work community as a source of strength in Appendix 1: Quote 17).

Paramedics wanted their work community and coworkers to know what they were going through. Even paramedics who changed jobs talked about their experiences in their new work communities. It was easier to work when their coworkers knew about what had happened and could take the lead in missions if needed. (For example, interviewee P was grateful that their experience was taken seriously, as they described in Appendix 1: Quote 18).

Interviewees pointed out that in EMS, colleagues often become close friends with whom they also spend leisure time. Interviewee O shared their feelings while in the sauna with colleagues.

*“And then during the holidays, well, we had a sauna with a couple of colleagues and took some leisure time, and with them, we discussed the incident thoroughly.”*

Many reflected on the culture of the profession and its potential for change to be more open about experienced feelings. They had been afraid of being belittled but were thankful that this did not happen. (For example, interviewee A wanted to be part of the culture change by openly discussing the issue, as they explained in Appendix 1: Quote 19).

### Left to cope alone

Some paramedics experienced bitter disappointment when they faced workplace violence and were left alone to deal with it. They felt that their employer’s attitude towards the incident was dismissive, or it was not recognized or managed appropriately at all. Interviewees felt that their employer lacked protocols on how to handle such situations. Interviewee I personally had to figure out the practical matters:

*“It was really sad to realize how alone the employer left us. Like we had to handle things ourselves, this coworker had connections with lawyers and others, so we figured things out ourselves, like contacting the unions and other things about how to handle it and how […] But yeah, very, very much alone, and then we realized that the employer didn’t have any system—like if something like this happens, then contact these people, or here are these numbers and these numbers, or these people and these people will help you or anything, so we had to deal with the aftermath pretty much by ourselves. That was a bit crappy.”*

Interviewee G described being belittled:

*“In that situation, there was, there was such an attitude. The attitude of the environment, a belittling attitude, even from the supervisory level.”*

In the shock phase after the incident, paramedics may not have even understood what had happened. There was a significant barrier to seeking help, such as a defusing session, if the employer did not offer it. Some paramedics also recognized that their inexperience was part of the reason they did not seek help; with their current understanding, they would demand help. (For example, interviewee R criticized the high threshold for seeking help and showing weakness in Appendix 1: Quote 20).

Even clear cases of violence and assault were sometimes dismissed, both by the employer and the police. Interviewees felt disbelief and a sense of unfairness. (For example, interviewee Q also felt belittled and questioned whether they had made a mistake in the situation, as they described in Appendix 1: Quote 21).

Paramedics wanted not only support from their employer but also reflection on what had happened and confirmation that they had acted correctly. Some of the interviewed paramedics also expressed concern for their coworkers. They worried that their coworker might be left alone with their thoughts. Interviewee R tried to support their coworker:

*“But then I did send many messages to my coworker ‘cause I didn’t know what their situation was, like if they had anyone to talk to.”*

### Permanent changes to work routines

The interviewed paramedics’ work patterns have undergone changes after experiencing workplace violence. Especially at the beginning, but even now, similar assignments or mission codes (especially mental health-related missions) cause anxiety and the thought of needing to be careful. (For example, interviewee A described their inner change in Appendix 1: Quote 22).

Interviewees described that they are more cautious when approaching the scene and take extra care to ensure their escape routes. Interviewee R has changed their work habits in many ways:

*“So, and it has remained to this day, that I always try to call [to the patient at] the scene, that has stayed. Yeah, escape routes. I have such a bad sense of direction, too, so I noticed that I used to be really lost at the scene when I had to leave. Like, where have we come from? So nowadays, I do notice that I pay more attention. Like making notes that here is this door, I will come out this way or something else that I didn’t even pay attention to before, but now I always do it to get out of there. I always tell my coworkers to turn the car around and always try to drive so that we can get out of there. So, I might not have thought about it that much before, but after that, I definitely do.”*

The interviewees also described that they do not enter the scene if they have any doubts, and they do not rely on a sense of security. (For example, naivety about the invincibility of paramedics has disappeared, as interviewee M described in Appendix 1: Quote 23).

### Resulting in professional growth

Experiencing workplace violence led many paramedics not only to change their operating procedures but also to professional growth. The interviewees shared how they can now read patients and situations better due to the development of their skills. (For example, interviewee A described their methods in Appendix 1: Quote 24).

Some paramedics were able and willing to harness their traumatic experience for positive use. They became determined to seek change and wanted to raise the issue for discussion. Interviewee F analyzed their recovery:

*“Then, perhaps, I’ve reached a point where I can transfer my own learning experiences related to this, even though they are unpleasant, to my colleagues, as I do a lot of supervision and communication, communication, and so on. I can use it elsewhere, too, and now, for example, I am training our summer employees and going through our tactical models or mini-TEMS action models and such things, so it has been helpful in that sense. I have been able to harness that to it, and I can make use of that.*

*Per se, I don’t like the saying ‘what doesn’t kill you makes you stronger’ because, in my opinion, it’s totally wrong because what doesn’t kill you can potentially cause serious harm. So, I don’t like those hero stories. But maybe by nature, I’m analytical enough to manage to find certain kinds of patterns or structures that can then maybe be transferred to my own actions; maybe it’s about problem-solving and the ability to change perspectives on some things.”* (Author comment: TEMS means Tactical Emergency Medical Services.)

Benefiting from their own experiences was an essential part of the recovery process and helped paramedics move forward. (For example, interviewee O started developing threat and violence training in their region, as they explained in Appendix 1: Quote 25).

### Eternal crack in the shell

The traumatic experience has left a lasting mark on some of the interviewees, and they feel that they carry the incident with them wherever they go. The incident has been a significant moment in their careers, and it continues to affect various aspects of their work. Interviewees were left to wonder about the effects of their psychological immunity being violated, as they, for instance, began having feelings of fear for their safety at work. (For example, interviewee I pondered switching careers in Appendix 1: Quote 26).

The incident may not be actively on their minds, but they described it as something that can resurface due to specific triggers. (For example, interviewee L described the lasting memories left by the incident, which still come to the surface occasionally, even years after the incident, as they described in Appendix 1: Quote 27).

Interviewee J recovered well otherwise, but the traumatic incident still occasionally surfaces in their dreams:

*“Unfortunately, even now, X years later, maybe 2–3 times a year, this issue comes up in nightmares. It’s not the actual person in the dream, but it’s just like a chasing situation where someone is trying to kill me. I’ve noticed that it’s only been a few weeks since I woke up from a nightmare like this, and it’s the same kind of chasing situation that was present in that situation X years ago. I believe it’s related to that incident because I’ve never had such nightmares before.”* (Author comment: The exact year was replaced with X due to privacy.)

Although the paramedics emphasized their recovery from the incident, discussing the topic still occasionally stirs up emotions. The role of family and the idea of starting a family also emerged in the interviews. Interviewee E contemplated how, in the future, they need to consider more than just their well-being:

*“And, of course, I’ve been thinking a lot afterward about, like now that I have this long parental leave here, what it will feel like when I return after this long time, and then that there’s already a child here, in a way, and there are other things to think about besides my own life, in a sense.”*

## Discussion

This study aimed to describe what kind of recovery processes paramedics have had after encountering violence during work. Ten recovery processes were identified. Recovery processes were not mutually exclusive, but they were sometimes sequential and overlapping, representing a phase of recovery that could be followed by another phase.

How paramedics react to WPV seemed to depend, for example, on their own experiences, work history, personal abilities, and factors that they could not describe. As they reported in this study, some incidents just “hit them from a different angle,” or they realized only afterward, confused, that a mission was bothering them [46]. This should be acknowledged, as one protocol may not serve them all, but individual aspects should be kept in mind when providing support for them after WPV.

After encountering WPV, paramedics require support from their organization/supervisors to help them cope with the incident [34, 39, 46, 47]. However, paramedics criticized the lack of protocols and support from their employer. It is not always clear to them if the organization has any protocols to deal with WPV [48] or if managers have the competence and willingness to help them [36]. Paramedics may hesitate to file a report about WPV, or in some cases, they wanted to, but their managers refused it [48]. In a Swedish study [14], paramedics also reported that they did not find filing a report to have any positive consequences on their safety or their recovery since even legal processes seldom led to actions against the perpetrator. However, keeping a record of WPV is crucial as it is the only way to collect data about their prevalence and effects and to convict perpetrators [14]. In many studies [47, 49], paramedics reported that they could not and would not talk with their managers or supervisors about their experiences and feelings regarding WPV, critical incidents, or mental distress in general. It is a shame, as the organization should be able to support its employees when it comes to something that has happened to them while on duty, as paramedics in this study pointed out. As stated, managers play a crucial role when it comes to the wellbeing of their employees after encountering critical incidents and WPV. The presence of managers is important, as it can lower the threshold for asking for help. Even more importantly, managers should create an atmosphere where employees feel that it is legitimate to ask for and receive help. In order that managers can make proper decisions and adjustments, they should have a thorough knowledge of their employees, which obviously is a challenge [50]. 

In this study, colleagues, professionals, friends, or family were reported to be among the most critical factors in helping paramedics in their recovery. A study by Tjin et al. [38] recognized the importance of first responders’ “trusted others,” those who were able to create psychologically safe spaces for talking and receiving emotional support and who promoted healthy coping mechanisms. They were also able to monitor first responders’ behavior and seek or offer professional help accordingly. Social support may help paramedics process the traumatic incident and ensure that they have acted correctly, thus reducing intrusive thoughts of “what ifs” [51], which were also noted in this study. In addition, social support reduces the likelihood of developing PTSD [36]. Social support from friends and family has been found to be the most important source of help in many studies [21, 49]. Even so, there are problems concerning that since paramedics may also close off their loved ones due to trying to protect them from secondary trauma [51].

According to the findings of this study, paramedics reported having participated in defusing and other support sessions of many forms. Generally, they felt them to be beneficial, regardless of the form. Defusing and debriefing have been subjects of debate in terms of their effectiveness. In Finland, defusing is more used amongst emergency services, and the difference from debriefing is that defusing sessions are not as intense or strictly structured [31]. Psychological debriefing has not been reported to prevent the development of PTSD symptoms or PTSD diagnosis, and it is not suggested to be used on a routine basis [52], but according to an assessment by Tamrakar et al. (2019), debriefing might be an effective intervention mechanism when used for its intended target group (emergency personnel and first responders) [53]. For emergency personnel, it might be useful for promoting recovery, helping paramedics return to normal functioning, and screening for individuals who may require further help when delivered within the correct timeframe (48–72 h) and by a trained professional [53]. Formal peer support or defusing sessions have been associated with lower levels of distress [47], increased feelings of resilience, and more positive attitudes towards expressing emotions [54]. However, in a recent Finnish study [55], defusing sessions were not associated with the occurring levels of secondary traumatic stress or burnout amongst paramedics.

In this study, formal types of support, such as sessions with a psychologist, were quite common forms of help. Paramedics found the help somewhat acceptable, but there was also concern regarding the suitability of the help, which is in line with previous studies [36, 56]. Service providers may lack paramedic-specific content knowledge and understanding of the pre-hospital work field. Moreover, In Finland, the content of the occupational health service paid by employers is slightly different between organizations [57]. Thus, the occupational health service may not cover enough psychologist visits for the paramedics to actually recover. Recent studies [31, 58, 59] conducted in Finland have demonstrated the impacts of attending a Post Critical Incident Seminar (PCIS); a support intervention aimed at emergency service personnel who have encountered critical incidents, such as WPV. The PCIS was originally developed for police officers, and participants are provided psychoeducation, peer support and therapeutic services [31]. Participating in PCIS may decrease symptoms of traumatic stress, depression and anxiety [58] and help participants improve their coping skills [59].

The culture of hardiness in EMS, which is widely recognized in previous studies [24, 36, 46, 47, 56, 60, 61], was brought up in many recovery processes. This culture is somewhat at odds with the culture of creating psychologically safe environment, in which there is, for example, permission to speak up and ask for help [62]. This culture of hardiness can appear, for example, as a belittling attitude or as thoughts that paramedics cannot show emotions. In the current study, some paramedics reported they felt the culture was changing, and they also wanted to change it themselves by talking about their feelings openly. However, others were victims of this culture, as they did not receive support or found that there was a significant barrier to asking for help. This culture is among the reasons why WPV is underreported and why paramedics refuse to receive help; they may fear being judged by their managers or colleagues as showing weakness and suffering from mental illness is seen as highly stigmatized within organizations [24, 36, 56, 60]. This culture is particularly harmful to young, inexperienced paramedics, who need peer support and experienced role models [38, 62] in order to develop healthy working habits and coping mechanisms. A supportive work environment, especially after critical incidents, seems to be related to paramedics’ well-being [47], which was also noted in this study. Creating a psychologically safe working environment has been seen as beneficial in terms of employees’ general wellbeing as well as the organizations’ ability to perform effectively [63].

WPV has been recognized to cause several negative consequences for working life, including the intent to quit the profession or change employers [23, 27, 64] and decreases workforce participation in general [27, 65]. Thus, WPV has substantial effects both for the employee and the organization, also causing financial challenges, for example, due to employees’ absence from work [27, 28, 34, 64, 65]. In Finland, as well as in many other countries, the employer has the statutory obligation to provide necessary measures of prevention and management of all threats, including WPV, towards their employees [66, 67]. Nonetheless, due to the nature of paramedics´ work field, it is not reasonable to assume that WPW could be wholly extinguished. A strict zero violence policy should be introduced in all healthcare sectors [25, 28, 29, 39, 64, 68], although it does not solve the problem completely, as patients or people accompanying them can always act unexpectedly. Organizations announcing a zero violence policy towards any kind of violent or aggressive behavior might send an important message, informing employees about the significance of their well-being and the organization’s and managers’ commitment to it [28, 29, 68]. Nonetheless, a zero violence policy can also be seen as unrealistic and impractical [26]. More broadly, organizations should leverage data accumulated from past experiences to prevent new incidents [69] and create a positive violence prevention climate where preventing WPV is of high importance throughout the organization and employees are proactively encouraged, for example, to report all incidents and to avoid behaviors that would trigger anger in others [50, 70]. There is evidence that such a climate reduces the occurrence of WPV [70, 71].

As our findings in this study highlight, attention should be paid to ensuring that all incidents involving violence or threats of it are processed with sufficient resources and in a way that the individual aspect is kept in mind. Some people have a delayed reaction, which is one of the reasons why, for example, McFarlane et al. (2009) suggest creating and keeping an incident register [72]. Screening of symptoms immediately after exposure may lead to missing crucial signs, but with a register, it would be possible to show causation afterward. The systematic registration of incidents is important to be able to gain knowledge of the issue and learn from the incidents, as recommended by ILO (2001) [73] and OSHA (2016) [74]. Using the collected data to analyze and observe different patterns and root causes and then develop prevention strategies accordingly is called data-driven prevention, which was demonstrated to be highly effective in a study by Arnezt et al. (2017) [69].

Moreover, Johnston et al. [35] suggest introducing mandatory time for employees to talk about the impact of work-related stress—this would be useful in terms of screening incidents of less severity, recognizing the potential cumulative burden and giving “permission” to talk. There are also other actions that organizations can and should take to ensure the well-being of paramedics. These actions should include paying attention to cultural shifts and organizational redesign of procedures, which means, for instance, removing the stigma associated with seeking help and adjusting staffing and rosters to ensure the well-being of staff [36]. Multiple studies suggested arranging education for paramedics to help them recognize their psychological distress and stress better [21, 36]. Organizations should also allow paramedics enough time to recover after encountering critical incidents [46, 47], which could be as little as a few hours to talk and rest. In summary, evidence shows that paramedics would greatly benefit from receiving more substantial support, empathy, and help from their employers and supervisors after encountering WPV [21, 34, 35, 46, 47].

### Strengths and limitations

This study was performed as a qualitative design with a narrative approach, which enables us to understand this phenomenon in depth in a context-specific setting but does not aim for broad generalizations [75]. The Finnish code of conduct for research integrity [44] was carefully followed during the whole process. The study’s trustworthiness was assessed using the criteria of credibility, dependability, confirmability, and transferability which were defined by Lincoln and Guba (1985) [76].

Two researchers from different backgrounds conducted this study. The first author is an experienced advanced-level paramedic familiar with WPV. This is both a strength and a limitation of this study. The first author´s pre-understanding of this subject was beneficial during the interviews, as it helped to gain the participants’ trust and to fully understand their language, thus enabling and increasing the credibility of this research. The last author is an experienced senior researcher with a background in occupational healthcare, disaster medicine, and pedagogy. Her research interests have mainly focused on the perspectives of work well-being among EMS personnel and the functionality of the emergency care system. Both authors worked closely together during the whole process. The first author strictly maintained her self-awareness throughout the interview process to avoid adding any preconceptions.

Dependability was strengthened by following a structured research plan and carefully documenting the entire research process. The narrative approach strengthened credibility, as it allowed paramedics to speak about the subject in interviews on their own terms, thus creating narratives that were not predetermined [40, 41]. However, the participants were interviewed only once, and they did not read the data gathered, which may be a limitation for the credibility. Yet, the data can be considered as saturated, as after 16 interviews, no new perspectives on recovery processes were found to emerge.

It is important to note that the type of violent incident was not asked during the interviews. It was considered more relevant that paramedics recognized that they had encountered WPV or threat in their work, and it had had an impact on them. This might be a general limitation because the recovery processes might vary based on the type of the incident (for example, verbal threat or physical attack).

The credibility of the study was strengthened by rereading and cross-checking the transcribed materials and repeatedly discussing and developing the analysis between the two authors to ensure mutual agreement was achieved. It is yet important to note that the narratives in data were not always temporally precise and sequential, as the recovery processes sometimes had several simultaneous narratives on different areas of life. Moreover, the narratives were vivid in language and experiences, and the authors carefully preserved the original content throughout the whole analysis. Following these, the thematic analysis of the data was a demanding, time-consuming iterative process, and it is possible that despite careful attention, misunderstandings of the content may have occurred, impacting theme formation [43]. A member check could have increased the credibility of the results, allowing each participant to recognize their own story among the themes identified. Moreover, having an external researcher review the research process and analysis would have increased the dependability and confirmability. Related to this, several quotes are provided from the original transcribed interview data to illustrate the analysis and enhance especially confirmability.

The progress of the study has been reported in detail to enhance its dependability and replicability in other areas and settings. Transferability was enhanced by ensuring a good recruitment process (recruiting paramedics from different parts of Finland) and the quality of the study sample. The study sample represents Finnish paramedics from different parts of Finland, who differ in terms of age, work, experience, and gender. Indeed, it is to be noted that EMS is organized in various ways in different countries, which may affect the transferability of the results globally. However, the results may be transferable to EMS settings where the policies, practices and cultural aspects are similar to those of the participants describe in our study.

Moreover, there has been much research about WPW among healthcare workers and paramedics. Even so, this was the first study to utilize a narrative approach and focus on the recovery processes, which can undoubtedly be seen as a strength. Recovery processes that were identified in this study align with what might be expected based on the extensive research previously conducted [5–9, 11–14, 16, 17, 20–24, 34, 47, 56], which reinforces the study’s confirmability. Found recovery processes offer more in-depth depictions and new perspectives on the subject, and the results are encouraging for further studies.

## Conclusions

This narrative study identified ten recovery processes paramedics have had after encountering violence during work. The findings presented in-depth personal experiences and revealed many factors that affect paramedics’ recovery processes. Paramedics felt that the incident had had an impact on them on many levels, leaving its mark on their ways of behavior and such. Needed and received help emerged from the results. While for some, the help they receive is adequate, some paramedics struggle to get appropriate support, especially from their organization and supervisors. Paramedics wanted their experience to be recognized and their reactions to be accepted. Our findings suggest that clear protocols should be established to help paramedics recover after encountering WPV. An individual aspect should be kept in mind, as not everybody reacts the same way.

Further studies should focus on exploring methods for recognizing and screening paramedics who would benefit from more substantial support after encountering WPV and what are the actual benefits of different support mechanisms, such as defusing sessions, occupational health services, supervision of work, and sick leave. Also, it would be beneficial to explore further the personal factors that influence paramedics’ recovery process and how the organizations and managers could recognize and promote these, both in advance and after a WPV.

### Electronic supplementary material

Below is the link to the electronic supplementary material.

Supplementary Material 1

## Data Availability

The datasets generated and analyzed during the current study are not publicly available because they contain participant-identifying information and are sensitive, and participants were assured that the data would be used only by the two researchers.
