# Supplementary material for: The recovery processes among paramedics who encountered violence during work—a narrative interview study
Source: J Occup Med Toxicol. 2024 May 16;19:17. doi: 10.1186/s12995-024-00417-6 (PMC11097420; doi:10.1186/s12995-024-00417-6)
Supplement: Supplementary file 1 — Supplementary Material 1 [file 12995_2024_417_MOESM1_ESM.docx]

Appendix 1. Quotations that are referenced in the results section from the original interview material.

| Quote # | Participant | Quote from the original interview material |
| --- | --- | --- |
| 1 | M | "Well, at the beginning, there was fear before they managed to catch the perpetrator. It took probably 2–3 weeks before it became clear who it was, and they were apprehended. So, the nights were difficult, really difficult, and I was quite tired back then ... […] Don't remember much from that time ... something like, if someone asks about what happened at work or at home during that period, I just don´t recall it. Actually, I would say the first two weeks were like a complete blur or that I don't remember anything from that time. If you ask me what the weather was like or anything else, I have no idea or what I did during that time.” |
| 2 | C | "Well, it was like, when I was heading to work, maybe even the evening before, I started feeling like my heart was racing a bit, like my heart rate went up, and then there was this kind of pressure in my chest. And then, going to work, it was there, but when I got to work and went to see a granny or something similar, it sort of eased away there at work. But, you know, leaving for work was like the worst-case scenario.” (Author comment: in this context, “granny” refers to an older female patient.) |
| 3 | P | "... and it was like, all the time, I kept questioning why I was doing this job? Why, why would I want to expose myself to these kinds of incidents when there's a chance that someone could attack me and there's a real risk of me getting seriously hurt? I thought about this a lot, and at that point, I already had a spot at a school lined up. I was going on a study leave. And there, it kind of solidified my decision that I wanted to take that study leave and continue my education. That I kind of had the chance to move away from this, you know, from this frontline role, and develop my professional skills so I could work somewhere else and not have to face these situations anymore." |
| 4 | K | "But it raises many questions, you know. There is a little uncertainty, the need to really go through that situation in your mind quite thoroughly as if there could have been a different way to handle it. But it does help the situation quite a bit when you realize that no, there's no way it could have gone differently or it could have happened to anyone. It was just terrible luck that we were there. But if we hadn't been there, it would have happened to a colleague, and it would have been really shocking if a colleague had told that story." |
| 5 | F | "Well, I have pretty conflicting feelings about it. When you naturally go to help a person, and you've been called in to help, and then the person decides to react in that way or maybe doesn't decide but ends up reacting in some way. And, of course, I examined my actions a lot, wondering if there was something I could have done to cause that situation. I concluded that no, I've acted just as I should have and would continue to act the same way in the future. And, you know, for a person who is in a terribly confused state, life is all jumbled up, speaking about a different patient, they probably don't see things in quite the same way as I do." |
| 6 | R | "So, I guess I started to become fearful, like, what if something actually happened—you know, just that kind of situation, of course—a very threatening situation where you couldn't escape? But, you know, for a while, it bothered me, and maybe more so that I was afraid of that situation. If I ended up facing it again, how panicked I would become when questioned whether I had said the right things [to the emergency response center through the emergency alert] or something else. So, I just started to fear, like, What if I have to? What if I freeze up there, unable to say anything anymore? So, you begin to overthink what you were supposed to say at that moment." |
| 7 | B | "At that point, my coworker and I immediately started to debrief the situation together. We decided to review the whole incident step-by-step, just like in those simulation lessons at school, like, 'Hey, we had this patient case. What happened? What did we do?' So, we systematically went through it. I feel it was incredibly helpful; it helped us organize the incident." |
| 8 | J | "So I got in touch with occupational health services the day after, so two days after the incident, and I don't know how we were blessed with this amazing occupational health psychologist, but they really took care of me. And I went to talk to them multiple times about what happened. [..] I believe the incident would have left me with a lot more fear if there had not been that occupational health psychologist because they were an absolute treasure.  […] Then, when I went to see the occupational health psychologist, I was still a bit shaken up about what had happened, and at first, I downplayed it a bit, like, ‘Well, nothing really happened; there's nothing to worry about.’ But then, the occupational health psychologist recognized the trauma it had left me with, and they said, ‘You will come back.’ Not like, do you want to come? But, you will come. So, I did go back for, I think, four or five sessions. Four times, yeah." |
| 9 | C | "But then I noticed that it got under my skin in a way that I started experiencing mild physical symptoms of anxiety when going to work after that incident. My heart rate would go up, and I had this lump right here. So, I continued with the occupational health psychologist, and I think I went for three or four sessions. Then, we started EMDR therapy. I felt a significant benefit from it, even after the first session. It helped tremendously in my recovery. […] So, maybe in this recovery process, I think that I wouldn't have managed without professional help, even though the situation may not have been particularly threatening. It just somehow hit me from a different angle, and I couldn't pinpoint why only that incident had such an impact compared to all the other missions.  […] So, what I was, obliviously, happy about in terms of my recovery was that the occupational health psychologist kind of understood our field of emergency care and understood, and didn't need us to explain it first, what it´s like. We got further like we were on the same page, and that made me feel safer and all. And then what maybe surprised me the most during my recovery was how the eye movement therapy, how quickly it started to work and help." (Author comment: EMDR means eye movement desensitization and reprocessing.) |
| 10 | B | "But maybe my biggest fear that hit me at that point was, like, I told my supervisor that I must take a sick leave ‘cause  just a week earlier, I had a very ethically challenging mission. It also involved a threat of violence, but it was different from this latest incident. There, the burden was more related to ethical and moral questions, as well as my feelings of inadequacy. I felt that I had too much stress and I was too emotionally sensitive at that point, so I had to take a breathing break. I couldn't go back to work. Even though I had two days off, and then I was supposed to come back to work, I felt like I couldn't do it. It was just before May Day, and May Day can be quite hectic. Usually, those missions are related to substance abuse too, so it wouldn't have been good if something negative had happened there." |
| 11 | F | "But like dealing with all these stupid legal and technical details, I had to go through the incident again and had to revisit it from different angles, which surprisingly turned out to be quite stressful and exhausting. I wasn't really prepared for that, and nobody was, and none of my colleagues (who had gone through something similar or even the lawyer) could really anticipate it either. And actually, after the court ruling, of course, the perpetrator wasn't going to pay any compensation, so we applied for it from the state treasury. It took a few months, and when it finally came through, I felt like, okay, this is now resolved. It felt like I could finally complete all the paperwork and move on mentally as well." |
| 12 | H | "So, through my role, I had to carry out this post-incident defusing session. […] It was challenging because I had to postpone my own. While others could immediately start processing and analyzing the situation, I had to maintain kind of a bit of an outsider's perspective or stay outside the processing, so to speak. And well, right at the beginning, there was this pressure to address it. I noticed there was a need to talk about it, to defuse it, but my role as the facilitator didn't allow me to take on the role of someone who needed to defuse it. I had to remain outside and manage the situation.  […] Then, my own defusing session didn't happen until the fourth or fifth day. At that point, everyone else had already defused the situation. This [delay] created different challenges because it wasn´t … it didn't feel fair to bring the others back to live through it again for a third time outside of their defusing sessions.  […] But then the defusing session. Well, I organized it for others there. I knew the whole time that I should have the same right to defuse, and it was a reality there. But looking back, it was unfortunate that it happened that way. I believe that maybe it could have been organized somehow. |
| 13 | D | "The process itself started to unfold immediately because I have a background in dealing with acute psychological crises … it has been an area of interest for me, and I'm familiar with psychological first aid. And I don't remember ... I think I was already part of a defusing group at that time.  […] For me, the process began to unfold within my mind because I knew it had to be processed and dealt with.  […] My background and expertise in various professional fields greatly aided the rapid removal of this situation from the forefront of my mind. My understanding of psychosocial support, understanding, and the understanding of comprehension of stress reactions also helped it." |
| 14 | O | "I've had quite a few close calls. Like once, I was threatened with a firearm. Well, it was a long time ago, and a couple of times, I had to run away. But usually, it has been resolved through dialogue, through talking. I don't know if it would make a difference in that way. Somehow, though, I have my perspective and worldview." |
| 15 | Q | "Well, during that time on sick leave, I did things I enjoyed […] I tied many fishing flies and went fishing and stuff like that. Slowly but surely, my sleep pattern began to return to normal. Once I got past the worst of it, the shock of the situation, I started slowly to solve the situation with my hobbies and activities. I tried to think as little as possible about work and what was happening there." |
| 16 | D | "Then we filed a police report, and it was processed as a criminal offense for an illegal threat. The person involved didn't deny the incident, so they received a fine as their punishment. And it was significant for me because that person didn't get away with what happened." |
| 17 | C | "We have a really, really good work community where we genuinely take care of each other. We ask and check on each other, so it was a significant factor, maybe in terms of my recovery. I knew, even though it was distressing to go to work, and I had physical symptoms, I knew the workplace itself was a safe environment. I could get help if something significant were to happen." |
| 18 | P | "But then I always talked to every coworker or partner about this issue and how I had been in such a situation; we need to take it slow. And everyone took it seriously. Every coworker was like, of course, they would go. I had mainly male partners then, so they would take on that macho role even more, like, okay, I need to protect that lady there. But yeah. Honestly, I feel that openly and honestly talking about the issue helped a lot in my own process and recovery from this matter." |
| 19 | A | "And, well, it has also been the support of the work community in a way, and maybe that understanding, like when I openly talked about this kind of situation, so then somehow it has been really nice that I haven't been judged, that maybe this kind of change is happening in our profession, that we talk about these things. Maybe it has been my own thought, too, that I want to talk about these things because these kinds of things happen, and maybe before there was this culture of not talking about it, like that it's just part of the job and you must endure it. So maybe I wanted to change that kind of culture myself, that you really can and should definitely talk about these things in the work community. And I have noticed that it has been a good thing to talk about it." |
| 20 | R | "Everyone said afterward that there should have been, of course, a defusing session and that everyone should know how to demand [it]. Nevertheless, somehow, in our culture, many refuse it when it's offered, and there's still a significant barrier to actually going and requesting it, especially if it hasn't been offered at all. In our case, the EMS field supervisor had already gone to their office, probably to sleep. So, nobody dared to knock on the door and say, ‘Hey, now the situation is such that we need a defusing session.’ Fortunately, no more missions came in because neither of us would have been in working condition for sure.  […] Yeah, somehow, it was handled very poorly, and HaiPro was just instructed to fill it out. After that, nobody has talked about it. So, it's like that.  […] And, like, many say that you can go to occupational health services to talk, and it's low-threshold help and everything. But we didn't even have any information about how to contact them. So how much better would that low threshold be if workplaces had direct instructions like if you need help, call this number or go here? So, it's a daunting barrier to start asking your supervisor like, where could I go." (Author comment: HaiPro is an incident reporting system.) |
| 21 | Q | "We had to figure out everything ourselves, how to proceed from here and such. I didn't get any assistance from management. So, I had to go and inform the managers myself, for instance, and explain the situation, that this has happened, and we need to make a HaiPro report about it. The employer's reaction was, ‘Have you made a medical error in the field?’ That was the first question. So, I said directly, ‘The patient attacked me. Do these incidents not usually require a HaiPro report?’ So …. So, I´d say it didn't go smoothly according to the protocol regarding employee protection and such. So, I was left with a shitty taste in my mouth about this whole thing because the employer side and the EMS field supervisor didn't consider the matter at all." (Author comment: HaiPro is an incident reporting system.) |
| 22 | A | "But like, I remember, I used to get very anxious and panicky about those missions at the beginning, but it was probably more in my own head, and it usually lasted, well, before it always happened on the way to the scene. But then, when I was in the situation and was able to interpret and read the situation, it often resolved itself, and then it got better. So maybe it was a kind of struggle with my mind that I feared it beforehand. […] Like in the beginning, probably the first year was the hardest. […] But then, I have a good technique for it here. Like I just take a few deep breaths, and it usually dissipates after that." |
| 23 | M | "Well, in a way, I have become more cautious and attentive to my surroundings and potential threats. And I'm glad that we use those vests now; I don't have to constantly justify or listen to teasing because I used them quite a lot after that incident. I mean bulletproof vests. So, yes, a certain level of precision and awareness of the fact that anything could be waiting behind that door really increased after that incident. And, of course, when it comes to guiding students, as I mentor a lot of them, I've also emphasized the aspect of safety much, much more. Before, maybe I might have been a bit more carefree and relied on the status of being a paramedic, like on invincibility. I guess a certain kind of naivety has disappeared." |
| 24 | A | "So, I notice certain things that have remained from that violent incident, but I don't feel like it restricts my ability to do my job anyhow, or that I'm really fearful, or anything like that. Perhaps it's more about situational awareness and being able to anticipate, always staying a step or two ahead in that situation. And you can think in your head, ‘What if something happens here? What will I do? Which direction will I go?’ So, it's like being able to process these things at the same time mentally." |
| 25 | O | "We got to, like, after that case, we started to push forward with workplace safety and other aspects. It was kind of a pivotal incident, so it helped in my own recovery because I felt like I could influence things, that it can't happen like this. We really need to start thinking about training for entering and exiting a scene and what kind of support the employer provides us in case of workplace violence. And now that we've made progress in these areas, we have threat and violence training in our region. […] We've covered topics such as legislation, situational exercises, verbal judo, and things like that. But it is; it should be an ongoing process in the future. For me, it was like a tool to start working on and improving things. I have a bit of (hidden to protect the interviewee's identity) in a good way, so looking ahead, I have a better feeling about it." |
| 26 | I | "To be honest, I've been contemplating a career change quite a lot, away from being a paramedic, even though it's been the coolest job in the world, and I can't pinpoint exactly why I don't feel it's for me anymore. But it could be, if I had to name some things, one could be the protection of my own mental health. I might not want to expose my mind to distressing things, dangerous things, or dangerous or distressing situations anymore. It's something like that." |
| 27 | L | "Well, I personally feel that I can't forget. I feel I can´t forget it because it was the first time and hopefully the last time something like that happened, and then, well. Yeah. I don't think I'll ever forget it ‘cause it was … there are so many memories, things that have stuck with me. And also, due to the adrenaline, it left a certain kind of memory trace. I don't believe it will ever fade away. It was, in my opinion, well ... I don't know if I can call it unique, but it's something you very rarely ever encounter. […] And then, if certain sounds that were present during the incident, if I hear them now, it takes me back to that moment for a second and reminds me that, ’Hey, this sound was there then,’ which intensifies the feeling." |
